# Phase and amplitude electroencephalography correlations change with disease progression in people with idiopathic rapid eye-movement sleep behavior disorder

**DOI:** 10.1093/sleep/zsab232

**Published:** 2021-09-22

**Authors:** Monica Roascio, Andrea Canessa, Rosella Trò, Pietro Mattioli, Francesco Famà, Laura Giorgetti, Nicola Girtler, Beatrice Orso, Silvia Morbelli, Flavio Nobili, Dario Arnaldi, Gabriele Arnulfo

**Affiliations:** 1 Department of Informatics, Bioengineering, Robotics and System engineering (DIBRIS), University of Genoa, Genoa, Italy; 2 Clinical Neurology, Department of Neurosciences, Rehabilitation, Ophthalmology, Genetics, Maternal and Children’s Sciences (DINOGMI), University of Genoa, Genoa, Italy; 3 IRCCS Ospedale Policlinico San Martino, Genoa, Italy; 4 Nuclear Medicine, Department of Health Sciences (DISSAL), University of Genoa, Genoa, Italy; 5 Neuroscience Center, Helsinki Institute of Life Science (HiLife), University of Helsinki, Helsinki, Finland

**Keywords:** cognitive impairment, rapid eye-movement sleep behavior disorder, hypersynchronization

## Abstract

**Study Objectives:**

Increased phase synchronization in electroencephalography (EEG) bands might reflect the activation of compensatory mechanisms of cognitive decline in people with neurodegenerative diseases. Here, we investigated whether altered large-scale couplings of brain oscillations could be linked to the balancing of cognitive decline in a longitudinal cohort of people with idiopathic rapid eye-movement sleep behavior disorder (iRBD).

**Methods:**

We analyzed 18 patients (17 males, 69.7 ± 7.5 years) with iRBD undergoing high-density EEG (HD-EEG), presynaptic dopaminergic imaging, and clinical and neuropsychological (NPS) assessments at two time points (time interval 24.2 ± 5.9 months). We thus quantified the HD-EEG power distribution, orthogonalized amplitude correlation, and weighted phase-lag index at both time points and correlated them with clinical, NPS, and imaging data.

**Results:**

Four patients phenoconverted at follow-up (three cases of parkinsonism and one of dementia). At the group level, NPS scores decreased over time, without reaching statistical significance. However, alpha phase synchronization increased and delta amplitude correlations decreased significantly at follow-up compared to baseline. Both large-scale network connectivity metrics were significantly correlated with NPS scores but not with sleep quality indices or presynaptic dopaminergic imaging data.

**Conclusions:**

These results suggest that increased alpha phase synchronization and reduced delta amplitude correlation may be considered electrophysiological signs of an active compensatory mechanism of cognitive impairment in people with iRBD. Large-scale functional modifications may be helpful biomarkers in the characterization of prodromal stages of alpha-synucleinopathies.

**Statement of Significance**

Cognitive impairment and rapid eye-movement sleep behavior disorder (RBD) emerge much earlier than the motor symptoms distinctive of alpha-synucleinopathies. For the first time, this study longitudinally investigated a group of people with idiopathic RBD (iRBD) who underwent high-density electroencephalography, presynaptic dopaminergic imaging, and comprehensive neuropsychological assessments at two time points. We found that large-scale network couplings were significantly correlated with cognitive data, possibly reflecting a compensatory mechanism for cognitive impairment. Advanced large-scale network investigations can increase the understanding of disease progression in alpha-synucleinopathies beginning in the prodromal stages. Larger studies with more than two time points can clarify the clinical trajectories of iRBD patients over time. By exploiting multimodal approaches, large-scale networks could be used to identify patients at high risk of short-term phenoconversion.

## Introduction

Rapid eye-movement sleep behavior disorder (RBD) is a parasomnia disorder that involves violent and undesirable behaviors [1] such as physical reactions to dreams due to the loss of normal muscle atonia during rapid eye movement (REM) sleep. RBD can occur alone (idiopathic RBD—iRBD) or in association with other neurological disorders (symptomatic RBD) [2], and people with iRBD have a high risk of developing Parkinson’s disease (PD), dementia with Lewy bodies (DLB), and multiple systemic atrophy [3, 4].

Clinical RBD symptoms include cognitive decline [4], impaired olfaction and color discrimination [5], abnormal metabolic network activity [6], and reduced striatal dopamine transporter (DAT) uptake [3].

Compared to healthy subjects, people with RBD show a reduction in dominant alpha rhythms in the occipital, parietal, and temporal lobes [7] along with power increases in the delta and theta frequency ranges in the frontal and central lobes [8–10]. In magnetoencephalographic studies, increases in theta, alpha, and beta phase synchronization have been associated with a compensatory mechanism for cognitive decline in mild cognitive impairment (MCI) [11, 12]. In MCI, hypersynchrony in the theta and beta bands has also been interpreted as a sign of the neurodegenerative process, where a loss of inhibitory neurons is caused by amyloid toxicity [13]. We hypothesized that hypersynchronization and altered amplitude coupling could be common features in the prodromal stages of several neurodegenerative diseases. However, it remains unclear how such electrophysiological biomarkers evolve over time in prodromal synucleinopathies. Here, we used a high-density electroencephalography (HD-EEG) system to investigate disease progression-related evolution in large-scale brain network couplings in a group of people with iRBD, and we correlated findings with longitudinal cognitive and nigrostriatal dopaminergic single-photon emission tomography (SPECT) data.

## Methods

### Data acquisition

A total of 22 people with iRBD (21 men; mean age 70 ± 6.8 years at the first clinical evaluation) were recruited at the sleep outpatient facility of the University Neurology Clinics at Policlinico San Martino in Genoa, and they were investigated in two subsequent sessions, namely, at baseline and at follow-up (after 24.2 ± 5.9 months; range: 14–41 months), including clinical, neuropsychological (NPS), EEG, and DAT-SPECT assessments. The diagnosis of idiopathic RBD was made according to international criteria (ICSD 3) and was confirmed by overnight video-polysomnography. In accordance with the Declaration of Helsinki, all participants gave informed consent before entering the study, which was approved by the local ethics committee.

#### Clinical assessment

 The patients underwent general and neurological examinations to exclude other neurological and psychiatric disorders. Brain magnetic resonance imaging (MRI), or computed tomography in the case MRI was unfeasible, was used to rule out brain diseases such as tumors or lesions. The presence of white matter hyperintensities was not an exclusion criterion if the Wahlund scale was not >1 for each brain region [14]. Patients underwent baseline clinical evaluations, including (1) the Mini-Mental State Examination (MMSE) as a global measure of cognitive impairment; (2) the Movement Disorder Society-sponsored revision of the Unified Parkinson’s Disease Rating Scale, motor section (MDS-UPDRS-III) to evaluate the presence of parkinsonian signs; (3) clinical interviews and questionnaires for activities of daily living (ADL) and instrumental ADL to exclude dementia; (4) the Beck depression inventory II (BDI-II) to rate depressive symptoms; and (5) the Italian version of Parkinson Disease Sleep Scale version 2 (PDSS-2) for a clinical measure of sleep disorders [15].

#### NPS assessment

Patients underwent a comprehensive NPS assessment, evaluating the five main NPS domains: language, executive functions, visuospatial abilities, memory, attention, and working memory. We administered the following NPS tests: semantic verbal fluency and phonemic verbal fluency, Stroop color word, Stroop color, Trail making tests A and B, clock completion, constructional apraxia, simple copy and copy with guiding landmarks, Rey Auditory Verbal Memory Test (immediate and delayed recall), Babcock story, Corsi span, digit span, and symbol digit. References and normative data are detailed in a previous study [16]. To minimize multicollinearity and to reduce the number of NPS variables for further statistical analysis, factor analysis with varimax rotation was applied to the baseline native NPS measures to identify the variables expressing a similar part of the total variance. We set a conventional threshold of 0.4 to factor loadings (expressing the factor-variable correlation) to select the group of variables mainly represented by each factor. Factor analysis identified four principal factors (Table 1 and Supplementary Table S1). Factor one was mainly related to visuospatial abilities (NPS-VS), factor two to verbal memory (NPS-VM), factor three to executive functions (NPS-EX), and factor four to attention (NPS-AT).

**Table 1.** Main clinical and demographic data of iRBD patients and baseline and follow-up

	Baseline	Follow-up
Age, years	69.7 ±7.5	71.7 ±7.5
MMSE	28.2 ±1.6	28.0 ±1.6
MDS-UPDRS-III	1.6 ±2.4	5.5 ±6.8
BDI-II	11.6 ±7.2	14.3 ±18.6
PDSS-2	18.9 ±7.7	17.5 ±13.9
NPS-VS	0.10 ±0.92	−0.10 ±1.02
NPS-VM	0.06 ±0.88	−0.06 ±1.07
NPS-EX	0.00 ±1.01	0.00 ±0.94
NPS-AT	0.07 ±0.88	−0.07 ±1.04
SBR Putamen	2.56 ±1.03	2.41 ±1.05
SBR Caudate	3.28 ±1.05	3.20 ±0.95

BDI-II, Beck depression inventory II; MDS-UPDRS-III, Movement Disorder Society-Unified Parkinson’s Disease Rating Scale, motor section; MMSE, Mini-Mental State Examination; NPS-VS, NPS-VM, NPS-EX and NPS-AT, neuropsychological visuospatial, verbal-memory, executive index and attention-mix, respectively; SBR, specific to non-displaceable binding ratio; PDSS-2, Parkinson’s Disease Sleep Scale, version 2.

#### Electrophysiological signal recording

All people with iRBD underwent HD-EEG evaluation during relaxed wakefulness within 3 months from diagnosis, late in the morning to minimize drowsiness. For each session, the acquisition protocol consisted of approximately 22.57 ± 2.62 min (range: minimum 17, maximum 29) of resting-state subdivided into 1.03 ± 0.67 min (range: minimum 0, maximum 3) with eyes-open, 3.8 ± 0.97 min (range: minimum 0, maximum 5) during hyperventilation and 17.75 ± 2.93 min (range: minimum 12, maximum 26) min with eyes closed. We used the Galileo system (EBNeuro, Florence, IT) to acquire bandpassed (0.3–100 Hz) signals from 64 electrodes at a sampling rate of 512 Hz. Electrodes were placed according to the 10–10 International System, where the reference electrode and ground were Fpz and Oz, respectively. We simultaneously recorded a horizontal electrooculogram to monitor eye movements with the same EEG recording parameters. Electrode impedances were closely monitored and kept below 5 kOhm. An EEG technician monitored the recording session to maintain a constant level of vigilance over the patient, prevent sleep and preserve a high signal quality across the whole recording session. For each patient and for both visits, we selected the eyes-closed condition for further analyses.

As a control dataset, we recruited 10 healthy people (9 men; mean age 68.6 ± 12.1 years) at Policlinico San Martino. The acquisition system and protocol are the same as those used to record people with RBD.

#### Molecular imaging evaluation

Within 3 months following diagnosis, all people with iRBD underwent [^123^I]N-ω-fluoropropyl-2β-carbomethoxy-3β-(4-iodophenyl)nortropane SPECT to measure the striatal dopamine reuptake transporter (DAT) density according to European Association of Nuclear Medicine (EANM) guidelines [17]. Details on SPECT data acquisition and analysis can be found in the Supplementary Materials. Reconstructed images were exported in the Analyze file format and processed by Basal Ganglia V2 software [18] to compute specific to non-displaceable binding ratios (SBRs). In particular, background uptake (occipital region) was subtracted from putamen or caudate uptake as follows: (putamen/caudate uptake—background uptake)/background uptake to compute SBR values. For subsequent analyses, we computed the mean SBR values between the right and left hemispheres for both the caudate and putamen.

### Data preprocessing

Data preprocessing and analysis were carried out in MATLAB, R2019a, using Brainstorm [19] and custom scripts.

As a first step, we preprocessed the EEG data of people with iRBD. We used a bandpass finite impulse response filter (1–80 Hz, Kaiser window, order 1858) to eliminate the low- and high-frequency artifacts, and we applied a zero-phase infinite impulse response notch filter (order 2) to remove the power line noise (50 Hz).

Later, we removed all channels and time windows showing artifactual activity, such as blinks, muscular activity, and other sporadic artifacts, using a combination of visual inspection and independent component analysis (ICA)-based rejection. In particular, for all subjects, we rejected by default channels A1, A2, and POZ since the percentage of artifactual windows was higher than 90% due to bad contact between the scalp and the electrode. We thus removed an average of 4.58 channels from each patient (range: minimum 0, maximum 8), and we interpolated those bad channels using spline interpolation (kernel size: 4 cm). Moreover, we rejected the time windows that showed evidence of drowsiness defined as those time intervals in which slow-wave activity replaced the occipital alpha rhythm.

Four subjects were excluded from the successive analysis due to excessive artifactual activity, which left less than 3 min of pruned eye closed resting-state data after cleaning. The final population size for this study was 18 patients.

We preprocessed the EEG time series of healthy subjects following the same procedure. We rejected channels A1, A2, and POZ by default, we removed an average of 3.7 channels (range: minimum 2, maximum 6), and we interpolated the bad channels.

For all cleaned datasets, we transformed to referenced EEG to scalp current densities (SCDs) [20, 21] for all clean sensors with the spline method (lambda 0.00001, order 4, degree 14). We defined five regions of interest for the subsequent analyses by grouping electrode contacts in the frontal, central, occipital, parietal, and temporal regions in accordance with their position on the scalp.

### Spectral analysis

We computed the power spectral density (PSD) for all channels and for all sessions in both the subjects with iRBD and healthy control subjects. The spectral analysis was performed using the Welch modified periodogram method (pwelch.m, MATLAB) between 1 and 30 Hz, with a resolution of 1 Hz. We computed the absolute power (AP) and relative power (RP) for four frequency bands of interest (BOIs) corresponding to common brain rhythms, that is, δ (1–4 Hz), θ (5–7 Hz), α (8–13 Hz), and β (13–30 Hz).

For each BOI *b*, the AP is computed as


$$\begin{array}{l} AP\left(b\right)=\frac{1}{|b|}\underset{f\in b}{\sum }\;P(\;f), \end{array}$$

where P(f) is the grand average power computed as the PSD average across all the channels.

Once the AP is computed, the RP of each BOI *b* is defined accordingly as:


$$\begin{array}{l} RP\left(b\right)=\;\;\;\frac{AP(b)}{AP(B)} \end{array}$$

where AP(B) is the AP computed over the whole frequency range considered B (1–30 Hz).

The simultaneous presence of an aperiodic background with a 1/f decaying spectrum superimposed on the true oscillatory periodic components characterizes the EEG recordings. The changes in the slope of the 1/f background activity could be representative of aging and, if pronounced, suggest an ongoing pathological degeneration process [22]. To properly analyze specific power changes, we decided to parameterize the estimated PSD. In this work, we modeled the PSD as the sum of putative, periodic oscillatory components parameterized by their central frequency, power, and bandwidth, as measured from Gaussian mixture model fits, plus an aperiodic component, as described by a power law fit *f*^*−*α^ [22]. Following the parameterization procedure, we computed the slope α of the aperiodic component to observe whether there was any difference between the two recording sessions.

### Phase and amplitude correlation analyses

We computed phase synchronization and amplitude correlations across a range of frequencies for all channel pairs and for all sessions in both subjects with iRBD and healthy control subjects. We analyzed the broadband SCD time series by means of a time-frequency decomposition using 15 narrowband Morlet wavelets in the 1–15 Hz range with 3-s time widths at 1 Hz [23].

Volume conduction, signal mixing, and source leakage significantly inflate phase synchronization analyses of sensor EEG data [24, 25] and reduce the possibility of detecting true significant changes in phase synchronization profiles. To minimize this, we adopted the weighted phase-lag index [24] (wPLI), as it is insensitive to volume conduction and has increased statistical power [24, 25] compared to other phase synchronization metrics.

The wPLI is defined as


$$\begin{array}{l} wPLI=\;\;\;\frac{|\;\;\;E\left\{I\left\{X\right\}\right\}\;\;\;|}{E\left\{\left|I\left\{X\right\}\right|\right\}}=\;\;\;\frac{|\;\;\;E\{\left|I\left\{X\right\}\right|\;\;\;sgn\left\{I\left\{X\right\}\right\}\}\;\;\;|}{E\{\left|I\left\{X\right\}\right|\}} \end{array}$$

where *I*{*X*} denotes the imaginary part and *X* is the cross-spectrum between two EEG channels.

While phase synchronization reflects a mechanism of neuronal communication per se, [26] coherent amplitude modulation between distant brain regions could reflect the concurrent activation of different neuronal populations in response to the same sensory stimulation. Hence, amplitude correlation could be seen as an electrophysiological correlate of functional connectivity analyses of blood oxygenation level-dependent (BOLD) signals. As in phase synchronization, volume conduction and source leakage mask true significant coupling in amplitude correlation analyses. As a volume conduction-insensitive measure, we adopted the orthogonalized Correlation Coefficient (oCC), which is defined as the Pearson correlation coefficient between two orthogonalized time series [27]. We computed oCC as:


$$\begin{array}{l} oCC\;\;\;=\frac{\overset{N}{\underset{k=1}{\sum }}X_{abs}\left(k\right)Y_{abs⊥X}\left(k\right)-N{\bar{X}}_{abs}{\bar{Y}}_{abs⊥X}}{(N-1)\sigma_{X_{abs}}\sigma_{Y_{abs⊥X}}} \end{array}$$

Where *X*_abs_ is the absolute value of the analyzed complex time series with standard deviation $\sigma_{X_{abs}}$ and mean value ${\bar{X}}_{abs}$, $Y_{abs⊥X}\;$is the absolute value of the complex signal Y(t) orthogonalized to the complex signal X(t), with its respective standard deviation $\sigma_{Y_{abs⊥X}}$ and mean value ${\bar{Y}}_{abs⊥X}$, N denotes the time series, and *k* is the sample number.

Both wPLI and oCC are limited in the [0,1] range, where 0 represents the absence of phase synchronization or amplitude correlation.

### Statistical analysis

First, we conducted a statistical analysis to investigate the cognitive decline in the subjects with iRBD and their neurodegeneration process. We applied the Kolmogorov–Smirnov test (kstest.m, MATLAB) to observe whether clinical, NPS, and imaging assessments came from a standard normal distribution. Given the absence of normality, we used the nonparametric Wilcoxon test (ranksum.m, MATLAB) to investigate whether clinical, NPS, and DAT-SPECT SBRs showed significant changes over time between baseline and follow-up.

Later, we wanted to test for systematic differences in EEG power between the two recording sessions in the subjects with iRBD. We thus applied a two-way repeated measures analysis of variance (rm_anova2.m, MATLAB, File Exchange) [28] to evaluate the statistical effects of channel groups and between the two recording sessions in AP, RP, and in the slope values for four main brain rhythms, namely, delta, theta, alpha, and beta. These multiple tests increase the false discovery rate, and we corrected all these *p* values with the Benjamini–Hochberg (BH) method (fdr_bh.m, MATLAB, File Exchange) [29]. Under the null hypothesis of no coupling, both wPLI and oCC show a Gaussian distribution with mean μ and variance σ ^2^ equal to the mean and the variance of the surrogates. Hence, for each pair of channels and a given frequency, we obtained a surrogate by arbitrarily rotating the time series of the second channel in one pair, and we computed the wPLI and oCC metric between the original signal of the first channel and the rotated version of the second channel. This operation yields a total of 3,600 surrogate pairs for each frequency. To quantify the threshold of significance, we first computed the null-H0 distribution parameters by calculating the sample mean and sample variance across surrogates. Then, we *z*-scored the metrics (i.e. wPLI or oCC) and used a *z*-score above 2.33 as the significance threshold of 0.02. To quantify the spatial extent of phase synchronization and amplitude correlations, we computed the fraction of significant edges (*z* > 2.33) for both subjects with iRBD and healthy control subjects. The fraction of significant is defined as the ratio between the number of statistically significant channel pairs out of the total number of channel pairs.

We investigated the difference in the wPLI and the oCC between the two recording sessions of subjects with iRBD to assess putative disease progression effects on large-scale brain networks. We wanted to observe whether the mean difference in the observed wPLI/oCC data was statistically significant. As a first step, we thus applied a multiple comparison permutation test [30] to calculate surrogate data to fix a threshold of significance for the difference. To account for autocorrelation in bivariate connectivity analyses, we mixed data between sessions at the level of a single subject (i.e. mixing edges across conditions) and then averaged within subjects and ultimately across subjects.

In particular, we computed surrogate data mixing the values (i.e. single-subject wPLI or oCC values) of observed data in the two recording sessions. To do so, we split the mixed values into two new vectors (one for each recording session) with the same dimensionality of two original categories. We thus computed the difference between the two new vectors for each subject, and we repeated this procedure 1,000 times. Since different data selection could yield an unbalanced variance between permutations during multiple permutations, we applied normalization. This standard procedure encompasses the normalization for each frequency by dividing each permutation by the global standard deviation computed across permutations [30]. Finally, for each permutated value, we selected the absolute maximum of the normalized surrogates across frequencies (max T-statistics multiple comparison correction). We defined the upper and lower bounds of confidence limits for the difference as the 2.5 and 97.5 percentiles across permutations. The mean difference between the observed data at baseline and follow-up was considered statistically significant when was outside the threshold range (*p* < .05). Given the limited size of our cohort, we adopted a more conservative approach, and we considered a significant effect on the difference between baseline and follow-up only those frequency points where both the confidence limits of the population variance (bootstrap, *N* = 1,000) were outside the threshold range.

As a last statistical analysis, we investigated whether there was a correlation between the altered phase synchronization and amplitude correlation with clinical, NPS, and DAT-SPECT data in subjects with RBD. Hence, we applied the partial Pearson correlation coefficient (partialcorr.m, MATLAB) to these data, controlling for the age of the patients at baseline and follow-up. Finally, we corrected the *p* values with the BH method (fdr_bh.m, MATLAB, File Exchange) [29] for multiple hypothesis testing.

## Results

After 24.4 ± 6.1 months of follow-up, four patients (22%) developed neurodegenerative diseases (three cases of PD and one case of DLB). As preliminary results, clinical, NPS, and DAT-SPECT data (Table 1 and Supplementary Table S2 for single subject details) did not show significant differences between baseline and follow-up.

### Effects of disease progression on amplitude spectral profiles

**Figure 1.**
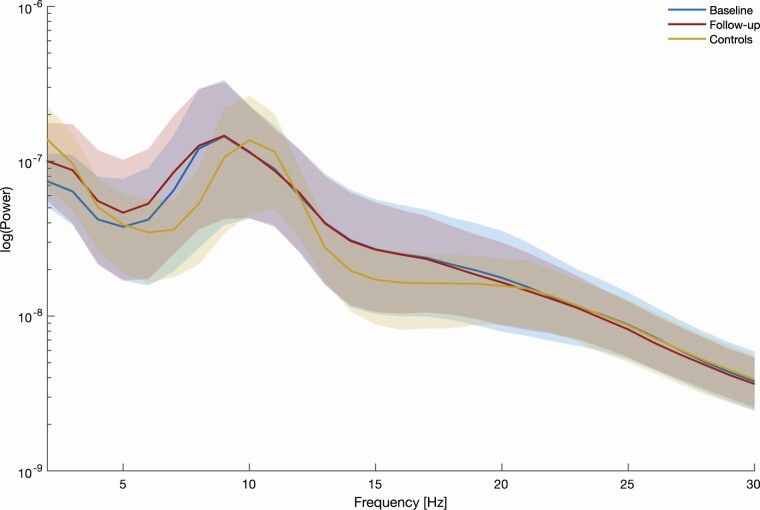
Effects of disease progression in power spectral profiles. Group-level averaged power spectral densities for subjects with RBD at baseline (blue), RBD at follow-up (red), and control group (yellow). Shaded areas represent confidence intervals at 5% around population mean (bootstrap, *N* = 1,000).

We tested whether the alpha band peak slowed as the disease progressed [7]. First, we observed that the overall power spectrum distribution of subjects with iRBD (at baseline and at follow-up) slows down toward a lower frequency compared to healthy control subjects (Figure 1), and indeed, recordings at both time points show a slowing of brain activity as measured by a RP increase in the theta band (5–7 Hz) and a reduction in the alpha (8–13 Hz) band compared to healthy control subjects.

Globally, the PSD did not differ between the two recording sessions in subjects with iRBD (Figure 1). Then, we investigated for a possible effect within the channel group (frontal, central, parietal, occipital, and temporal). For each channel group and each recording condition of the subjects with iRBD, we evaluated the RP (Supplementary Figure S1a–e and Table S3) and the AP (Supplementary Figure S1f–j and Table S5). RP (Supplementary Table S3) and AP (Supplementary Table S5) showed significant differences across channels in all frequency bands (two-way repeated measures ANOVA with BH correction), except for RP in the theta band. This is an expected effect, which confirms the quality of the recorded data. We observed no significant difference between recording sessions or their interaction with channel groups in RP and AP values. No other factors or their combinations reached statistical significance in terms of the AP and RP values.

Moreover, we extracted the scaling exponent of fitted power laws and evaluated whether there was a significant difference between recording sessions or EEG channels. Additionally, in this case, we did not observe a significant difference between recording sessions or their interaction with channel groups (Supplementary Table S4).

Our results confirm that people with iRBD show a slowing of the power spectrum toward lower frequencies compared to healthy subjects, but there is no significant difference in power spectrum or in its slope in the time span over which we observed disease progression.

### Global phase synchronization and amplitude correlation change with disease progression

**Figure 2.**
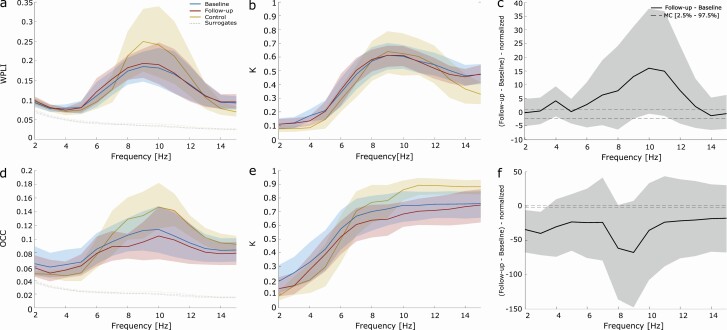
Global phase synchronization and amplitude correlation change with the disease progression. (A, D) Strength and (B, E) extent of population averaged wPLI (A, B) and oCC (D, E) at baseline (blue), at follow-up (red) and in healthy control subjects (yellow). Shaded areas represent confidence intervals around population mean (bootstrap, *N* = 1,000). Dashed lines represent surrogate averages. (C, F) Absolute difference of normalized phase synchronization (C) and normalized amplitude correlation (F) spectral profiles between conditions. Shaded areas represent confidence intervals at 5% around mean (bootstrap, *N* = 1,000). Dashed lines represent significance threshold for a two-tail pairwise permutation test at (*p* < .05) corrected for multiple comparison using max T. statistics across frequencies. wPLI, weighted phase-lag index; oCC, orthogonalized Correlation Coefficient; MC, multiple comparison; *k*, fraction of significant.

We then investigated how phase synchronization and amplitude correlation changed between baseline and follow-up. First, we observe group differences for the strength (Figure 2A) and spatial extent (Figure 2B) of global wPLI for both recording sessions and within all channel groups (Supplementary Figure S2a–e). In detail, alpha band (10 Hz) wPLI decreased in subjects with iRBD compared to controls (Figure 2A), and it significantly increased (*p* < .05, permutation test − max T. corrected for multiple comparison) in subjects with iRBD at the second visit (Figure 2C). The increment could also be observed within channel groups except for the frontal and temporal channel groups (Supplementary Figure S2a–e). Moreover, the large-scale amplitude correlation strength (Figure 2D), but not spatial extent (Figure 2E), was significantly (*p* < .05, permutation test − max T. corrected) reduced from the first to the second recording session in the delta (4 Hz) band (Figure 2F) and in all channel groups (Supplementary Figure S2f–j). Altogether, these results highlight the global functional modifications associated with disease progression in subjects with iRBD.

### Large-scale network modifications related to disease progression

We looked at the organization of the resulting large-scale brain networks and asked whether specific groups of channels were more affected as the disease progressed.

**Figure 3.**
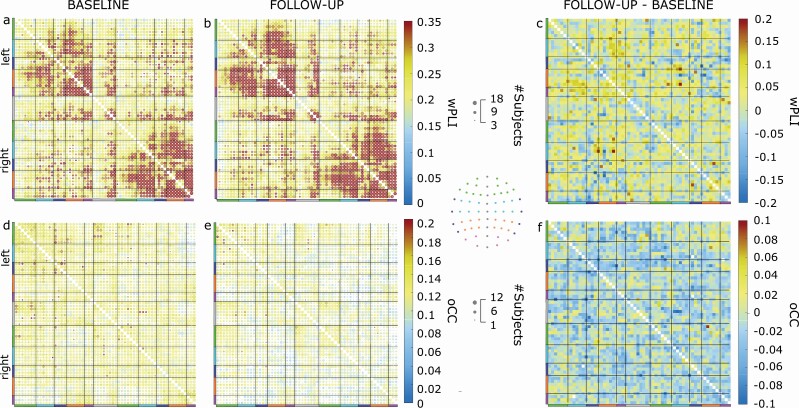
Large-scale network modifications related to disease progression. Bubble chart of population averaged wPLI (A, B) in alpha band (10 Hz) and oCC (D, E) in delta band (4 Hz) at baseline (A, D) and at follow-up (B, E) for significant edges (*p* < .02). The color bar represents the wPLI or OCC values. The bubble size is proportional to the number of subjects having that edge as a significant edge. The colored lines represent the channel group: frontal (green), central (azure), temporal (purple), parietal (orange), occipital (pink), and midline (gray). The topography shows the position of the channels for each channel group. (C) Difference of wPLI values between follow-up and baseline at 10 Hz and (F) difference of oCC at 4 Hz. wPLI, weighted phase-lag index; oCC, orthogonalized Correlation Coefficient.

Phase synchronization and amplitude correlation matrices can be interpreted as adjacency matrices that represent a weighted undirected graph. A weighted graph is a mathematical object defined by nodes and edges connecting two nodes. Here, each channel represents a node, and an edge connecting two nodes is weighted by the relative wPLI or oCC values. We represented the adjacency matrices using bubble graphs where color intensity and bubble size are proportional to metric value and the number of subjects having that edge as significant (Figure 3). We focused on the two frequency bands showing the most significant differences, namely, alpha (10 Hz) and delta (4 Hz), in wPLI and oCC, respectively. We quantified the group-level wPLI and oCC matrices averaging all edge values across subjects for a given visit. Phase synchronization increases from baseline to follow-up, and this increase is linked to edges that are significant in more than 3 and in up to 18 patients (Figure 3A–C). The amplitude correlation decreased from baseline to follow-up, and this decrease was linked to edges that were significant in up to 12 patients (Figure 3D–F).

These results suggest that the increase in phase synchronization (Figure 3C) and the reduction in amplitude correlation (Figure 3F) do not affect specific brain areas but rather are large-scale phenomena.

### Phase synchronization and amplitude correlation values correlate with clinical and NPS scores

**Figure 4.**
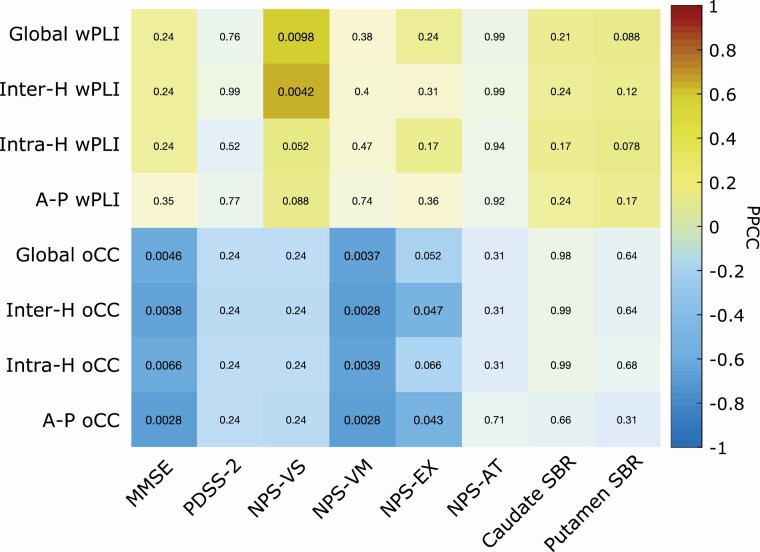
Phase synchronization and amplitude correlation values correlate with clinical and NPS scores. Age-adjusted partial Pearson correlation coefficient with Benjamini–Hochberg correction of wPLI (10 Hz) and oCC (4 Hz) with clinical, cognitive, and mean SBR data. The color represents the correlation values and the number in each box represents the false discovery rate (FDR) corrected *p* values. PPCC, partial Pearson correlation coefficient; H, hemispheric; A-P, Antero-Posterior; wPLI, weighted phase-lag index; oCC, orthogonalized Correlation Coefficient; MMSE, Mini-Mental State Examination; PDSS-2, Parkinson’s Disease Sleep Scale; NPS-VS, NPS-VM, NPS-EX, and NPS-AT, neuropsychological visuo-spatial, verbal-memory, executive index, and attention-mix, respectively; SBR, specific to non-displaceable binding ratio.

Finally, we asked whether phase synchronization and amplitude correlation values correlate with the clinical score, NPS indices and mean SBR data. First, we computed the age-adjusted partial Pearson correlation coefficient of clinical and NPS scores with wPLI at 10 Hz and oCC at 4 Hz (Figure 4), pooling subjects and visits. We observed a direct significant correlation between global and interhemispheric wPLI values and visuospatial NPS scores (*p* = .0098 and *p =* .0042, respectively; BH corrected). Additionally, oCC showed an inverse correlation with clinical and NPS scores. In particular, we observed significant negative correlations between oCC and MMSE (*p* = .0046 – global; *p =* .0038 – interhemispheric; *p* = .0066 – intrahemispheric; and *p* = .0028 antero-posterior oCC configuration; BH corrected), verbal memory (*p* = .0037 – global; *p* = .0028 – interhemispheric; and antero-posterior; *p* = .0039 – intrahemispheric oCC configuration; BH corrected) and executive index (*p* = .047 interhemispheric and *p* = .043 – antero-posterior oCC configuration; BH corrected).

## Discussion

In this study, for the first time, we longitudinally investigated people with iRBD by using serial HD-EEG, DAT-SPECT, and clinical and NPS assessments. Patients were investigated at the time of iRBD diagnosis and then approximately 2 years later. First, we found a consistent shift of the alpha power peak towards the theta band of subjects with iRDB compared to age-matched healthy controls (Figure 1). This is in agreement with previous literature data [7–10, 31, 32]. At the follow-up visit, the slowing of the main alpha rhythm remained stable without clear worsening as the disease progressed. However, by analyzing large-scale brain network coupling, we found a significant increase in phase synchronization in the alpha (8–13 Hz) band in each channel group (Figure 2A–C and Supplementary Figure S2a–e) from baseline to the follow-up session. A large body of evidence has shown the importance of studying phase synchronization in prodromal and early stages of neurodegenerative diseases [11–13, 33–36].

Hypersynchrony in the theta, alpha, and beta bands has been linked with disease progression [11, 13, 36] and decreases at advanced stages of pathology [13, 35]. On the one hand, the increase in phase synchronization was associated with the activation of a brain compensatory mechanism to control cognitive impairment [11]. On the other hand, hypersynchronization was considered a sign of system malfunctioning that anticipates the breakdown of the network in the dementia stage [13]. Studies on Alzheimer’s disease have shown that the deposition of amyloid-β yields a loss of excitation/inhibition balance, that is, a pathological sign of disease progression, and this imbalance was correlated with hypersynchronization in the MCI [37, 38].

Altered phase synchronization may also be observed in prodromal synucleinopathy. Indeed, putative hallmarks of cognitive decline and neurodegeneration include slowing of EEG alpha rhythms [7] and alterations of global and network-specific functional connectivity in both electrophysiological [39] and resting-state functional MRI [40–42]. In particular, in people with iRBD and cognitive impairment, the power of alpha rhythm decreases and is replaced by an increase in delta/theta activity compared to age-matched healthy controls [8]. In the present study, the values of the phase synchronization in the alpha band were significantly and positively correlated with the NPS scores (Figure 4), especially in visuospatial functions. This finding supports the hypothesis that a phase synchronization increase can reflect the activation of a compensatory mechanism of the brain to counterbalance cognitive decline during neurodegeneration in people with iRBD. It is also worth highlighting that the phase synchronization in the alpha band showed direct but not significant correlations (*p* = .11 – global, intrahemispheric and anteroposterior; *p* = .12 – interhemispheric wPLI configuration; BH corrected) with nigrostriatal dopaminergic function (Figure 4), particularly at the putamen level, which is a known marker of neurodegeneration in people with iRBD and in synucleinopathy in general.

As a further result, we found significant reductions in the amplitude correlation in all lobes at follow-up compared with the baseline (Figure 2D–F and Supplementary Figure S2f–j). In particular, this reduction was significant in the delta band (4 Hz) (Figure 2F) and was inversely and significantly correlated with MMSE, verbal memory, and executive functions (Figure 4). RBD symptoms are known to affect large-scale global and between-region resting-state networks. The moment-to-moment correlation of EEG amplitude fluctuations shows spatial patterns similar to BOLD resting-state networks [27]. Previous literature data found altered BOLD nigrostriatal, nigrocortical, and corticocortical activity in people with iRBD [40–43]. In particular, in agreement with our data, a disruption of functional connectivity in the posterior brain regions has been associated with cognitive impairment in people with iRBD [40].

Sleep disruption may play a role in cognitive function; [44] thus, we investigated whether large-scale functional couplings may be associated with sleep alteration. In our sample, we did not find any significant correlation between delta amplitude correlation or alpha phase synchronization and PDSS-2 scores (Figure 4); thus, we may speculate that the association between phase synchronization and amplitude correlation and cognitive function is independent of sleep disruption in our subjects with iRBD.

There are some limitations in this study. First, the small number of subjects involved limits the generalizability of our observations. This work constitutes a preliminary report of a larger project, the first of its kind, aiming at robustly investigating advanced electrophysiological markers in a longitudinal study of people with iRBD. Nonetheless, our results fit with the current understanding of phase synchronization modifications in neurodegenerative disease and represent the first report of longitudinal electrophysiological modifications in the prodromal stage of synucleinopathy. Second, volume conduction and signal mixing significantly inflate the phase synchronization and amplitude couplings. This effect is even larger for scalp recordings. For this reason, in the present work, we adopted state-of-the-art metrics able to limit such bias. Multiple sources of evidence showed that real zero-lag synchronization could be observed between distant cortical areas [45–47]. Adopting zero-lag insensitive metrics hence discards all those true components, possibly reducing the true measured effects.

The human brain changes over the human life span, and these physiological changes can become pathological modifications during neurodegenerative disease progression. One may argue that increased age at follow-up might confound our results since physiological age effects cannot be separated. Nonetheless, evidence suggests that phase synchronization decreases as a function of age [48]. Given that we measure an increase (rather than a decrease) between subsequent visits, this effect is unlikely to be due to aging alone. Unfortunately, the absence of a longitudinal study with healthy controls undermines our possibility of running a robust assessment.

Moreover, a large fraction of clinical observations are still based on scalp-level recordings, and our results suggest that scalp large-scale network changes can be a useful additional variable to study prodromal synucleinopathy. However, only two time points cannot be used to infer accurate model prediction for disease progression. We are actively working toward increasing the number of subjects involved and the number of follow-up visits.

In conclusion, this study, for the first time, investigates the changes in phase synchronization and amplitude correlation in subjects with iRBD at two distinct time points using HD-EEG and correlating the data with longitudinal DAT-SPECT and cognitive assessment. Understanding the coherent evolution over time of combined clinical, imaging and neurophysiological biomarkers in iRBD could improve our understanding of the prodromal stages of synucleinopathy, possibly helping in identifying those patients who are more likely eligible for neuroprotective treatment. Our results suggest that a global increase in alpha phase synchrony and a reduction in the delta amplitude correlation might represent an electrophysiological correlate of the activation of a large-scale compensatory mechanism to balance cognitive decline. However, we cannot directly eliminate a pathological role similar to what was observed in people with MCI.

## Supplementary Material

zsab232_suppl_Supplementary_Materials
