# Supplementary material for: Phase and amplitude electroencephalography correlations change with disease progression in people with idiopathic rapid eye-movement sleep behavior disorder
Source: Sleep. 2021 Sep 22;45(1):zsab232. doi: 10.1093/sleep/zsab232 (PMC8754497; doi:10.1093/sleep/zsab232)
Supplement: zsab232_suppl_Supplementary_Materials [file zsab232_suppl_supplementary_materials.pdf]

Phase and amplitude EEG correlations change with disease progression in people with idiopathic rapid eye-movement sleep behavior disorder

Roascio M.[1,*]; Canessa A.[1]; Trò R.D.[1]; Mattioli P.[3,4]; Famà F.[3,4]; Giorgetti L.[4]; Girtler N.[3,4]; Orso B.[3]; Morbelli S.[4,5]; Nobili F.M.[3,4]; Arnaldi D.[3,4,°]; Arnulfo G.[1,2,°]

[1] Department of Informatics, Bioengineering, Robotics and System engineering (DIBRIS), University of Genoa, Genoa, Italy.

[2] Neuroscience Center, Helsinki Institute of Life Science (HiLife), University of Helsinki, Helsinki, Finland

[3] Clinical Neurology, Department of Neurosciences, Rehabilitation, Ophthalmology, Genetics, Maternal and Children's Sciences (DINOGMI), University of Genoa, Genoa, Italy.

[4] IRCCS Ospedale Policlinico San Martino, Genoa, Italy

[5] Nuclear Medicine, Department of Health Sciences (DISSAL), University of Genoa, Genoa, Italy

* corresponding author: Monica Roascio, Via All'Opera Pia 13, Genoa, Italy, monica.roascio@edu.unige.it

° these authors equally contributed to this work

Supplementary Materials

^{123}I-FP-CIT-SPECT image acquisition and reconstruction.

To perform SPECT images, we used a dual-head Millennium VG camera (GE Healthcare) equipped with low energy, high resolution, parallel-beam collimators. Scans have been acquired 180-240 minutes after intravenous administration of 185 MBq of ^{123}I-FP-CIT (DaTSCAN, GE Healthcare, Little Chalfont, Buckinghamshire, UK), and lasted 40 minutes. We applied a "step-and-shoot" protocol with a radius rotation lower than 15 cm, and 120 projections evenly spaced over 360° were generated. Total counts were comprised between 2 and 3 million. We used an electronic zoom (zoom factor = 1.8) during data collecting phase to obtain an acquisition matrix's pixel size of 2.4 mm. Subsequently, a digital zoom was applied during the reconstruction phase. Resulting images were sampled by cubic voxels (2.33 mm). We processed projections by applying an OSEM algorithm (8 interactions, 10 subsets) that included a proback pair accounting for collimator blur and photon attenuation, and then post-filtering (3-D Gaussian filter with full-width at half maximum 58 mm). Photon attenuation was modelled with the approximation of a linear coefficient uniform inside the skull and equal to 0.11 cm^{-1}, and a 2D+ 1 approximation was applied in the simulation of the space simulation blur. Compensation for scatter was not performed.

Basal Ganglia software functioning.

Basal Ganglia software is an automatic algorithm based on a high-definition, 3-dimensional striatal template derived from the Talairach atlas. In detail, an automated algorithm performs fine adjustments in positioning blurred templates to match radioactive counts, meanwhile, it locates occipital regions of interest (ROI) to perform background evaluation. Also, a partial volume effect (PVE) correction is performed during the uptake computation of the putamen, caudate and background.

List of Supplementary Tables

Table S1**: **Factor analysis results. Factors and corresponding neuropsychological tests with their respective factor loading are shown. A conventional factor loading threshold of 0.4 was used. ***Legend.*** NPS-VS, NPS-VM, NPS-EX and NPS-AT = Neuropsychological visuo-spatial, verbal-memory, executive index and attention-mix, respectively; TMT= trail making test; C.A.= constructional apraxia; RAVLT= Rey Auditory Verbal Memory Test.

Table S2: Cognitive decline effects are not apparent with disease progression. On the rows, for each subjects, we observed the baseline (up) and the follow-up (bottom) values for each index. The two last row showed the mean ± standard deviation for both baseline and follow-up, and the p-value of the Wilcoxon test computed between baseline and follow-up values, respectively. * indicates the RBD patients which are evolved in PD at follow-up, while ** indicates the patients which are evolved in DLB. The PDSS-2 is estimated using an alternative method in baseline, marked with #. ***Legend.*** BDI-II= Beck Depression Inventory II; MDS-UPDRS-III= Movement Disorder Society-Unified Parkinson's Disease Rating scale, motor section; MMSE= Mini-Mental State Examination; NPS-VS, NPS-VM, NPS-EX and NPS-AT = Neuropsychological visuo-spatial, verbal-memory, executive index and attention-mix, respectively; SBR= Specific to non-displaceable binding ratio. PDSS-2 = Parkinson's Disease Sleep Scale; SD = Standard Deviation.

Table S3**: **Repeated Measure ANOVA two-ways over Relative Power values. *We* compared the mean difference between the recording session in each brain rhythm (delta, theta, alpha and beta).

Table S4: Repeated Measure ANOVA two-ways over slope alpha values. We compared the mean difference between the recording sessions.

Table S5**: **Repeated Measure ANOVA two-ways over Absolute Power values. We compared the mean difference between the recording sessions in each brain rhythm (delta, theta, alpha and beta).

List of Supplementary Figures

Figure S1**: **Disease progression effects on amplitude distributions. Raincloud plots of (a-e) relative power and (f-j) absolute power as a function of frequencies for all 5 channel groups: frontal (a,f), central (b,g), parietal (c,h), occipital (d,i) and temporal (e,j). Dots represent relative power of each single channel pooled across subjects at baseline (blue) and at follow-up (red).

Figure S2: Disease progression enhances the phase-synchronization in alpha band and reduces the amplitude correlation in delta*.** Strength of population averaged (a-e) weighted Phase Lag-Index (wPLI) and (f-j) orthogonalized Correlation Coefficient (oCC) at baseline (blue) and at follow-up (red) in each channel group: (a,f) frontal, (b,g) central (c,h) parietal, (d,i) occipital and (e,j) temporal. Shaded areas represent confidence interval around population mean (1000 bootstraps). Dashed lines represent surrogate average for each group. ***Legend. wPLI = weighted Phase Lag Index; oCC = orthogonalized Correlation Coefficient.

	NPS-VS	NPS-VM	NPS-EX	NPS-AT
Semantic verbal fluency	0.43			
Stroop Color	0.54			
TMT-A	-0.42			
Clock completion test	0.65			
C.A. simple copy	0.90			
C.A. guiding landmarks	0.94			
RAVLT, immediate recall		0.81		
RAVLT, delayed recall		0.99		
Babcock story		0.85		
Stroop Color Word			0.40	
Corsi span			1.02	
TMT-B			-0.70	
Symbol digit			0.56	
Phonemic verbal fluency				0.73
Digit span				0.97
**Variance explained (%)**	**63.6**	**13.2**	**10.1**	**7.9**

Table S1: ***Factor analysis results.*** *Factors and corresponding neuropsychological tests with their respective factor loading are shown. A conventional factor loading threshold of 0.4 was used.* ***Legend.*** *NPS-VS, NPS-VM, NPS-EX and NPS-AT = Neuropsychological visuo-spatial, verbal-memory, executive index and attention-mix, respectively; TMT= trail making test; C.A.= constructional apraxia; RAVLT= Rey Auditory Verbal Memory Test.*

	AGE	MMSE	PDSS-2	BDI-II	MDS-UPDRS-III	NPS-VS	NPS-VM	NPS-EX	NPS-AT	SBR Putamen	SBR Caudato
**S1**	69	28	15,41#	15	3	0,59	-0,16	0,27	0,57	1.585	3.125
	71	27	2	12	3	-0,44	-1,97	0,19	-0,42	0.705	2.575
**S2**	62	29	16,95#	2	0	1,2	-0,14	1,46	0,35	3.235	3.785
	64	30	7	3	0	1,23	0,44	1,45	-0,34	2.685	3.125
**S3****	77	24	10,70#	7	2	-1,87	-2,33	-2,69	-0,59	1.75	2.85
	79	25	6	5	3	-2,33	-2,29	-0,86	-1,5	1.42	2.135
**S4**	72	27	38	19	8	-0,028	-0,21	-0,49	0,45	2.795	3.73
	74	28	31	14	23	-0,48	0,22	-1,08	-0,23	3.345	5.16
**S5**	71	29	17,33#	12,6	0	-2,31	-0,58	-0,097	-1,78	2.745	3.56
	74	27	4	12	3	-1,75	-0,29	0,0096	-1,62	3.015	3675
**S6***	76	26	12,11#	29,4	3	-0,11	-0,74	-1,38	0,068	0.815	2.46
	78	25	-	10	15	-0,17	-0,83	-1,83	-1,03	0.815	2.19
**S7**	74	27	9	3	0	-0,27	0,46	-0,64	-1,5	4.225	5.49
	76	29	4	3	0	0,11	0,56	-0,29	-1,52	2.96	3.51
**S8**	71	26	12,30#	0	2	-0,19	-0,31	0,38	0,30	2.905	3.345
	74	27	-	6	6	0,25	0,14	1,11	1,08	2.85	3.015
**S9**	72	30	11	15	0	0,25	0,45	0,55	2,01	1.42	2.02
	73	28	-	25,2	10	-1,75	-0,045	-1,25	1,67	-	-
**S10**	74	29	17,97#	4	0	0,27	0,12	0,91	0,95	2.74	3.29
	77	28	20	9	0	0,091	0,64	0,49	0,91	2.905	3.62
**S11***	67	28	20,29#	16	4	0,25	1,2	0,15	0,59	1.035	1.145
	69	27	35	15	11	0,55	0,19	0,14	0,59	0.595	1.31
**S12**	60	29	24	17	0	0,62	0,77	-0,55	0,56	3.29	3.455
	63	30	25	9	0	0,64	0,58	0,299	0,64	3.125	3.895
**S13**	69	29	17,73#	10	0	0,25	0,95	-0,44	-0,22	2.35	2.63
	72	30	43	9	0	0,15	0,084	-0,33	-1,18	2.245	2.355
**S14**	77	30	17,45#	16	1	0,099	0,97	0,63	-0,092	2.85	3.29
	80	29	17	84	3	0,41	1,61	0,58	0,70	3.07	3.62
**S15***	60	29	19,65#	14	6	0,16	-0,30	-0,60	-0,34	2.19	2.52
	62	29	38	25	16	-0,57	-1,18	-0,91	-0,57	2.575	3.325
**S16**	82	30	34	13	0	0,97	1,45	0,39	0,0019	2.905	4.115
	84	29	15	11	0	0,86	1,28	0,48	1,17	-	-
**S17**	77	29	24	6	0	0,49	-0,36	0,63	0,68	2.3	2.905
	80	26	4	3	6	0,0040	-1,26	0,096	-0,44	1.97	3.29
**S18**	53	29	22	9	0	1,39	-0,24	1,55	-0,69	4.885	5.38
	55	30	11	3	0	1,45	1,10	1,67	0,86	4.28	4.445
**mean ± SD**	69,7 ± 7,5	28,2 ± 1,6	18,9 ± 7,7	11,6 ± 7,2	1,6 ± 2,4	0,098 ± 0,9	0,056 ± 0,9	0,002 ± 1,0	0,07 ± 0,9	3,3 ± 1,1	2,6 ± 1,0
	71.7 ± 7.5	28,0 ± 1,6	17,5 ± 13,9	11,0 ± 7,3	5,5 ± 6,8	-0,098 ± 1,0	-0,056 ± 1,1	-0,002 ± 0,9	-0,07 ± 1,0	3,2 ± 0,9	2,4 ± 1,0
p	n.a.	0.66	0,29	0,58	0,15	0,46	0,94	0,79	0,86	0.91	0.88

Table S2: ***Cognitive decline effects are not apparent with disease progression****. On the rows, for each subjects, we observed the baseline (up) and the follow-up (bottom) values for each index. The two last row showed the mean ± standard deviation for both baseline and follow-up, and the p-value of the Wilcoxon test computed between baseline and follow-up values, respectively. * indicates the RBD patients which are evolved in PD at follow-up, while ** indicates the patients which are evolved in DLB. The PDSS-2 is estimated using an alternative method in baseline, marked with #.* ***Legend.*** *BDI-II= Beck Depression Inventory II; MDS-UPDRS-III= Movement Disorder Society-Unified Parkinson's Disease Rating scale, motor section; MMSE= Mini-Mental State Examination; NPS-VS, NPS-VM, NPS-EX and NPS-AT = Neuropsychological visuo-spatial, verbal-memory, executive index and attention-mix, respectively; SBR= Specific to non-displaceable binding ratio. PDSS-2 = Parkinson's Disease Sleep Scale; SD = Standard Deviation*

	Delta Rhythm	Theta Rhythm	Alpha Rhythm	Beta Rhythm
**Recording sessions**	p = 0.85	p = 0.54	p = 0.96	p = 0.53
**Electrode groups**	$p = 7.2*10^{-8}$	p = 0.055	$p = 3*10^{-15}$	$p = 2.9*10^{-11}$
**Sessions x Groups**	p = 0.50	p = 0.055	p = 0.65	p = 0.055

Table S3: Repeated Measure ANOVA two-ways over Relative Power values. *We compared the mean difference between the recording session in each brain rhythm (delta, theta, alpha and beta).*

	$\alpha\ (1/f^{\alpha})$
**Recording sessions**	p = 0.87
**Electrode groups**	$p = 2.8*10^{-4}$
**Sessions x Groups**	p = 0.37

Table S4: Repeated Measure ANOVA two-ways over slope alpha values. *We compared the mean difference between the recording sessions.*

	Delta Rhythm	Theta Rhythm	Alpha Rhythm	Beta Rhythm
**Recording sessions**	p = 0.50	p = 0.31	p = 0.50	p = 0.68
**Electrode groups**	$p = 6.6*10^{-7}$	$p = 2.6*10^{-9}$	$p = 1.9*10^{-12}$	$p = 5.4*10^{-6}$
**Sessions x Groups**	p = 0.84	p = 0.50	p = 0.85	p = 0.85

Table S5: Repeated Measure ANOVA two-ways over Absolute Power values. *We compared the mean difference between the recording sessions in each brain rhythm (delta, theta, alpha and beta).*

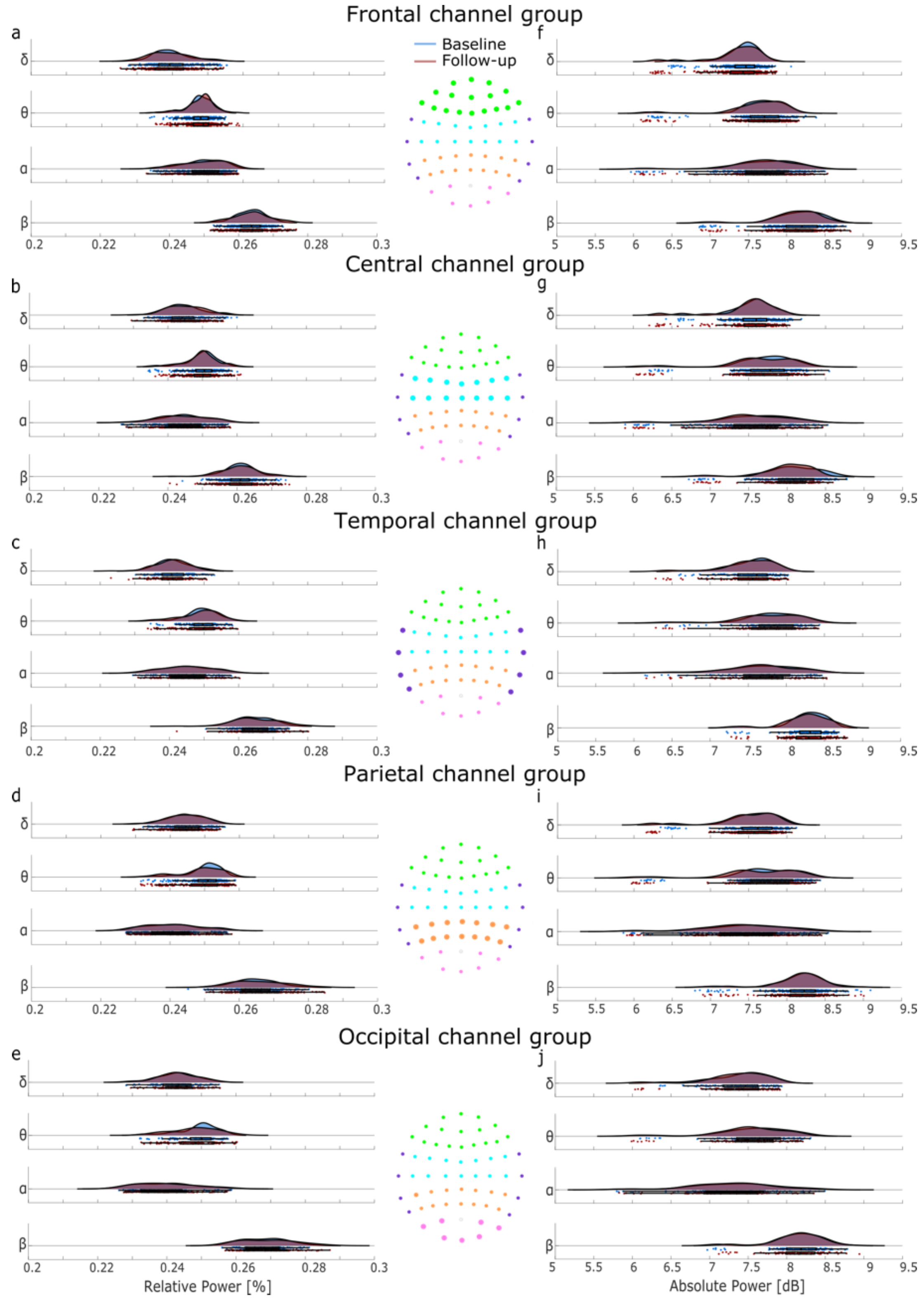

Figure S1: Disease progression effects on amplitude distributions. Raincloud plots of (a-e) relative power and (f-j) absolute power as a function of frequency bands for all channel groups: frontal (a,f), central (b,g), parietal (c,h), occipital (d,i), and temporal (e,j). Dots represent relative power of each single channel pooled across subjects at baseline (blue) and at follow-up (red).

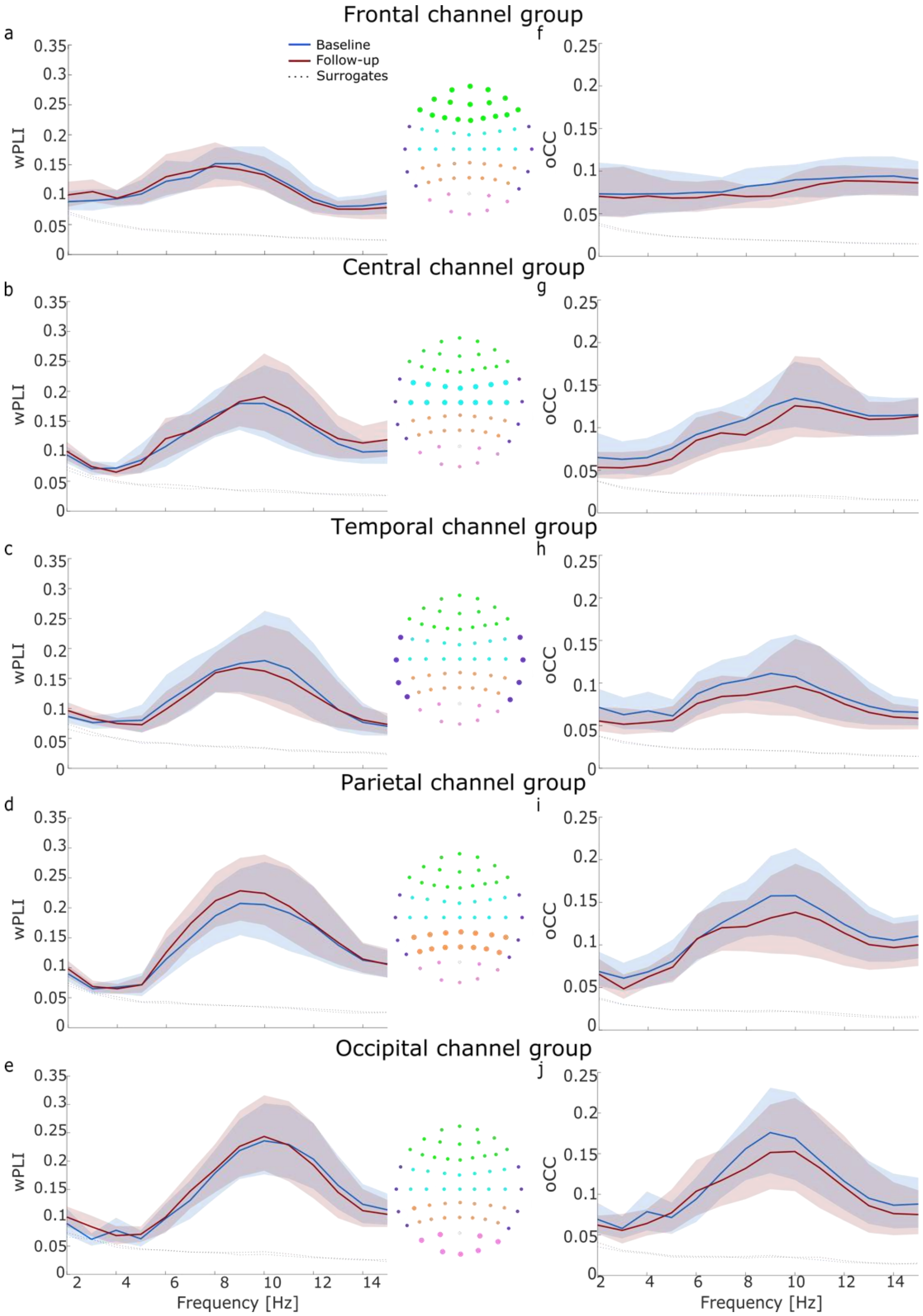

Figure S2: Disease progression enhances the phase-synchronization in alpha band and reduces the amplitude correlation in delta. Strength of population averaged (a-e) weighted Phase Lag-Index (wPLI) and (f-j) orthogonalized Correlation Coefficient (oCC) at baseline (blue) and at follow-up (red) in each channel group: (a,f) frontal, (b,g) central (c,h) parietal, (d,i) occipital, and (e,j) temporal. Shaded areas represent confidence interval around population mean (bootstrap, N = 1000). Dashed lines represent surrogate average for each group. Legend. wPLI = weighted Phase Lag Index; oCC = orthogonalized Correlation Coefficient.
